# Supplementary material for: A Likelihood Approach for Real-Time Calibration of Stochastic Compartmental Epidemic Models
Source: PLoS Comput Biol. 2017 Jan 17;13(1):e1005257. doi: 10.1371/journal.pcbi.1005257 (PMC5240920; doi:10.1371/journal.pcbi.1005257)
Supplement: S1 File — (TAR.GZ) [file pcbi.1005257.s014.tar.gz › HSPH_Online-SI-Revision/IPoi09/n10-mild/n10-mild_BENCH2_table4.pdf]

1	newly 3 weeks spec	153.	272.717	{130., 506.}	86.4348	0.236057	newly 3 weeks cumul	425.	961.113	{504., 1558.}	127.627	0.324479	newly 5 weeks spec	103.	191.358	{78., 414.}	99.194	0.251805	newly 5 weeks cumul	653.	1384.92	{677., 2426.}	114.514	0.291751	newly 46 weeks cumul	1470.	2095.11	{907., 3968.}	64.4243	0.202766
2	newly 3 weeks spec	110.	295.827	{131., 591.}	168.934	0.363679	newly 3 weeks cumul	464.	1183.79	{542., 2151.}	155.127	0.352985	newly 5 weeks spec	57.	153.115	{54., 329.}	171.39	0.360039	newly 5 weeks cumul	610.	1554.02	{690., 2911.}	154.776	0.348383	newly 51 weeks cumul	742.	1928.81	{826., 3761.}	159.973	0.352175
3	newly 3 weeks spec	211.	151.654	{60., 324.}	52.8436	0.303304	newly 3 weeks cumul	768.	715.269	{302., 1490.}	44.7065	0.206103	newly 5 weeks spec	97.	66.8846	{20., 140.}	56.3045	0.337291	newly 5 weeks cumul	973.	885.135	{360., 1864.}	46.0017	0.216658	newly 54 weeks cumul	1157.	1006.63	{413., 2108.}	47.0265	0.230726
4	newly 3 weeks spec	113.	415.269	{202., 752.}	267.494	0.523684	newly 3 weeks cumul	452.	1461.16	{846., 2225.}	224.429	0.486074	newly 5 weeks spec	81.	284.836	{125., 566.}	251.649	0.484832	newly 5 weeks cumul	619.	2092.	{1150., 3641.}	237.964	0.494778	newly 49 weeks cumul	827.	3248.99	{1558., 6186.}	292.864	0.5428
5	newly 3 weeks spec	93.	193.913	{107., 306.}	112.716	0.303652	newly 3 weeks cumul	276.	619.783	{364., 901.}	127.032	0.334626	newly 5 weeks spec	92.	166.891	{74., 282.}	90.5718	0.258383	newly 5 weeks cumul	472.	964.87	{531., 1443.}	108.355	0.297045	newly 29 weeks cumul	1568.	2309.35	{999., 4393.}	67.6353	0.21622
6	newly 3 weeks spec	108.	85.9268	{25., 137.}	52.8681	0.295006	newly 3 weeks cumul	406.	461.122	{203., 744.}	54.4155	0.212521	newly 5 weeks spec	80.	30.6098	{9., 52.}	69.5427	0.597548	newly 5 weeks cumul	611.	541.878	{240., 897.}	50.8642	0.253062	newly 55 weeks cumul	829.	580.976	{249., 944.}	51.2254	0.30717
7	newly 3 weeks spec	89.	199.673	{63., 382.}	138.386	0.314529	newly 3 weeks cumul	391.	837.531	{280., 1571.}	124.067	0.30393	newly 5 weeks spec	87.	105.245	{27., 185.}	74.8299	0.281366	newly 5 weeks cumul	552.	1089.04	{354., 1989.}	110.703	0.278385	newly 51 weeks cumul	801.	1314.49	{417., 2390.}	85.9156	0.239756
8	newly 3 weeks spec	197.	313.267	{157., 515.}	67.9357	0.202737	newly 3 weeks cumul	615.	1102.87	{616., 1755.}	81.2629	0.23265	newly 5 weeks spec	164.	210.55	{99., 381.}	53.0386	0.183588	newly 5 weeks cumul	920.	1570.23	{847., 2561.}	74.8152	0.215428	newly 46 weeks cumul	2034.	2389.85	{1196., 3984.}	45.2368	0.169742
9	newly 3 weeks spec	116.	495.985	{256., 800.}	327.573	0.596007	newly 3 weeks cumul	592.	1712.57	{1037., 2450.}	189.285	0.438644	newly 5 weeks spec	85.	350.94	{165., 653.}	312.871	0.557113	newly 5 weeks cumul	760.	2486.16	{1408., 3880.}	227.127	0.485816	newly 50 weeks cumul	872.	3807.7	{1916., 6430.}	336.663	0.595278
10	newly 3 weeks spec	91.	117.184	{36., 242.}	63.3603	0.232998	newly 3 weeks cumul	345.	475.868	{184., 914.}	62.5706	0.209301	newly 5 weeks spec	65.	64.2632	{21., 117.}	49.1498	0.223111	newly 5 weeks cumul	482.	625.605	{242., 1182.}	58.3479	0.204533	newly 47 weeks cumul	1027.	796.947	{285., 1578.}	47.9424	0.269201
11	newly 3 weeks spec	165.	98.2632	{42., 191.}	52.9506	0.344513	newly 3 weeks cumul	495.	464.368	{210., 884.}	42.1159	0.195458	newly 5 weeks spec	56.	40.2105	{13., 88.}	51.5038	0.318832	newly 5 weeks cumul	648.	566.5	{248., 1099.}	43.5063	0.216921	newly 52 weeks cumul	801.	634.737	{283., 1235.}	44.1356	0.237309
12	newly 3 weeks spec	53.	177.115	{52., 405.}	236.901	0.371536	newly 3 weeks cumul	224.	826.131	{286., 1816.}	269.335	0.440553	newly 5 weeks spec	9.	75.9508	{20., 176.}	743.898	0.731898	newly 5 weeks cumul	264.	1018.11	{338., 2224.}	285.972	0.450304	newly 54 weeks cumul	276.	1143.05	{359., 2563.}	314.291	0.474855
13	newly 3 weeks spec	135.	377.4	{214., 610.}	182.357	0.43018	newly 3 weeks cumul	528.	1316.64	{787., 1981.}	153.833	0.396438	newly 5 weeks spec	51.	264.273	{149., 449.}	418.182	0.674557	newly 5 weeks cumul	676.	1905.16	{1112., 2970.}	184.384	0.434281	newly 46 weeks cumul	774.	3225.4	{1716., 4938.}	316.718	0.579641
14	newly 3 weeks spec	150.	300.609	{141., 516.}	103.865	0.269168	newly 3 weeks cumul	578.	1074.1	{526., 1795.}	89.647	0.246774	newly 5 weeks spec	106.	212.058	{75., 506.}	110.528	0.267748	newly 5 weeks cumul	857.	1539.25	{708., 2621.}	85.6493	0.237279	newly 47 weeks cumul	1338.	2440.88	{981., 5119.}	92.1546	0.236827
15	newly 3 weeks spec	208.	211.159	{75., 353.}	47.913	0.213853	newly 3 weeks cumul	656.	798.886	{346., 1361.}	48.2019	0.179097	newly 5 weeks spec	192.	122.864	{42., 220.}	50.8049	0.323558	newly 5 weeks cumul	1065.	1087.7	{448., 1861.}	42.9684	0.189978	newly 49 weeks cumul	2326.	1453.36	{576., 2417.}	48.4777	0.311221
16	newly 3 weeks spec	196.	338.554	{144., 531.}	82.6531	0.242227	newly 3 weeks cumul	619.	1301.8	{603., 2029.}	113.558	0.301812	newly 5 weeks spec	104.	197.677	{79., 333.}	99.6302	0.270401	newly 5 weeks cumul	881.	1762.25	{780., 2779.}	105.12	0.285794	newly 50 weeks cumul	1128.	2320.02	{974., 3776.}	110.806	0.292793
17	newly 3 weeks spec	103.	338.718	{199., 433.}	231.342	0.509311	newly 3 weeks cumul	286.	986.359	{493., 1285.}	248.055	0.527358	newly 5 weeks spec	112.	324.179	{222., 431.}	191.873	0.456026	newly 5 weeks cumul	495.	1636.67	{1106., 2047.}	233.468	0.515517	newly 47 weeks cumul	2645.	4297.18	{2534., 5929.}	64.6338	0.203917
18	newly 3 weeks spec	134.	207.162	{67., 440.}	81.1013	0.250917	newly 3 weeks cumul	498.	754.297	{273., 1542.}	71.9255	0.224802	newly 5 weeks spec	79.	132.243	{40., 319.}	100.445	0.285222	newly 5 weeks cumul	686.	1052.78	{367., 2218.}	76.5543	0.234963	newly 47 weeks cumul	921.	1486.54	{518., 3150.}	86.5364	0.249519
19	newly 3 weeks spec	74.	128.397	{45., 287.}	93.2665	0.232772	newly 3 weeks cumul	333.	566.121	{207., 1211.}	86.9162	0.223654	newly 5 weeks spec	49.	61.431	{20., 142.}	76.038	0.267206	newly 5 weeks cumul	449.	715.879	{267., 1551.}	80.8578	0.217547	newly 50 weeks cumul	666.	844.448	{311., 1845.}	67.1119	0.228658
20	newly 3 weeks spec	165.	204.136	{79., 365.}	62.562	0.224634	newly 3 weeks cumul	661.	862.386	{376., 1516.}	58.2141	0.199327	newly 5 weeks spec	141.	101.477	{34., 174.}	58.3656	0.344108	newly 5 weeks cumul	964.	1109.73	{457., 1975.}	54.7341	0.212134	newly 51 weeks cumul	1374.	1323.5	{524., 2405.}	51.5251	0.237635
21	newly 3 weeks spec	122.	262.889	{119., 468.}	120.613	0.288559	newly 3 weeks cumul	466.	1076.26	{517., 1842.}	133.445	0.320289	newly 5 weeks spec	62.	140.296	{58., 265.}	132.676	0.295618	newly 5 weeks cumul	595.	1409.57	{667., 2415.}	139.374	0.325216	newly 51 weeks cumul	738.	1751.31	{786., 3068.}	140.161	0.321212
22	newly 3 weeks spec	145.	263.5	{125., 463.}	91.0464	0.25156	newly 3 weeks cumul	458.	967.345	{470., 1686.}	116.014	0.303946	newly 5 weeks spec	86.	171.966	{63., 341.}	109.944	0.273684	newly 5 weeks cumul	631.	1351.64	{604., 2405.}	119.463	0.306913	newly 47 weeks cumul	1216.	1980.78	{783., 3555.}	76.5185	0.215294
23	newly 3 weeks spec	51.	202.711	{72., 294.}	297.647	0.495581	newly 3 weeks cumul	384.	824.556	{339., 1300.}	120.295	0.293027	newly 5 weeks spec	14.	114.667	{30., 134.}	719.048	0.720852	newly 5 weeks cumul	436.	1093.71	{415., 1656.}	154.297	0.33179	newly 52 weeks cumul	442.	1354.36	{477., 1889.}	208.074	0.387353
24	newly 3 weeks spec	109.	67.8293	{15., 156.}	82.0765	0.563085	newly 3 weeks cumul	313.	287.927	{81., 621.}	79.2021	0.382539	newly 5 weeks spec	56.	35.5366	{5., 75.}	86.8902	0.617072	newly 5 weeks cumul	432.	373.	{101., 780.}	81.3403	0.415343	newly 48 weeks cumul	566.	455.122	{109., 903.}	81.9357	0.447674
25	newly 3 weeks spec	134.	287.37	{98., 607.}	127.667	0.303364	newly 3 weeks cumul	550.	1242.35	{511., 2258.}	130.582	0.314524	newly 5 weeks spec	58.	131.148	{29., 260.}	148.595	0.329191	newly 5 weeks cumul	716.	1572.13	{591., 2960.}	126.57	0.303776	newly 55 weeks cumul	798.	1804.8	{634., 3385.}	134.069	0.311823
26	newly 3 weeks spec	59.	109.5	{43., 226.}	99.5094	0.236547	newly 3 weeks cumul	261.	437.447	{218., 962.}	79.3406	0.20725	newly 5 weeks spec	33.	64.3947	{23., 108.}	112.998	0.257459	newly 5 weeks cumul	353.	585.395	{281., 1243.}	80.4607	0.208907	newly 46 weeks cumul	475.	761.421	{349., 1531.}	80.2216	0.21038
27	newly 3 weeks spec	87.	417.881	{217., 671.}	380.323	0.644518	newly 3 weeks cumul	397.	1435.83	{836., 2161.}	261.67	0.530585	newly 5 weeks spec	24.	307.068	{117., 509.}	1179.45	1.04108	newly 5 weeks cumul	477.	2097.36	{1140., 3221.}	339.697	0.611714	newly 48 weeks cumul	499.	3430.47	{1586., 5537.}	587.47	0.78072
28	newly 3 weeks spec	95.	116.676	{32., 234.}	62.7596	0.246587	newly 3 weeks cumul	273.	377.459	{109., 746.}	69.5674	0.242657	newly 5 weeks spec	72.	92.7568	{26., 196.}	66.1411	0.248265	newly 5 weeks cumul	428.	573.811	{164., 1198.}	67.3971	0.241695	newly 26 weeks cumul	1195.	1170.19	{332., 2512.}	56.2253	0.273003
29	newly 3 weeks spec	140.	296.348	{124., 686.}	120.4	0.289864	newly 3 weeks cumul	520.	1114.08	{508., 2378.}	119.607	0.298649	newly 5 weeks spec	86.	180.	{53., 413.}	122.516	0.283765	newly 5 weeks cumul	706.	1525.91	{645., 3437.}	122.508	0.297995	newly 49 weeks cumul	1008.	2015.03	{801., 4568.}	109.301	0.268751
30	newly 3 weeks spec	99.	212.074	{89., 355.}	120.165	0.300527	newly 3 weeks cumul	453.	835.796	{375., 1336.}	92.9074	0.25822	newly 5 weeks spec	51.	123.111	{47., 196.}	146.333	0.336587	newly 5 weeks cumul	566.	1121.87	{488., 1848.}	105.127	0.278124	newly 48 weeks cumul	783.	1490.06	{642., 2501.}	99.3307	0.266416
31	newly 3 weeks spec	92.	147.975	{37., 302.}	99.4837	0.288864	newly 3 weeks cumul	415.	736.1	{228., 1575.}	103.193	0.276421	newly 5 weeks spec	49.	59.85	{10., 140.}	88.2653	0.371358	newly 5 weeks cumul	552.	890.825	{250., 1918.}	94.3614	0.274747	newly 55 weeks cumul	668.	973.575	{271., 2101.}	86.2013	0.277245
32	newly 3 weeks spec	124.	81.4737	{30., 164.}	50.8489	0.318231	newly 3 weeks cumul	386.	285.105	{121., 566.}	47.2321	0.271945	newly 5 weeks spec	137.	57.6579	{23., 111.}	62.2167	0.487516	newly 5 weeks cumul	648.	412.763	{171., 820.}	51.0843	0.323187	newly 28 weeks cumul	1971.	739.737	{276., 1450.}	66.5839	0.540389
33	newly 3 weeks spec	96.	392.225	{187., 670.}	308.568	0.56696	newly 3 weeks cumul	537.	1398.11	{760., 2194.}	161.725	0.390607	newly 5 weeks spec	49.	263.	{120., 472.}	436.735	0.669381	newly 5 weeks cumul	666.	1985.7	{1041., 3276.}	198.154	0.437892	newly 49 weeks cumul	775.	3028.83	{1473., 5326.}	290.817	0.539139
34	newly 3 weeks spec	217.	252.067	{95., 488.}	59.7542	0.23418	newly 3 weeks cumul	685.	941.8	{374., 1742.}	63.0316	0.210321	newly 5 weeks spec	140.	150.983	{48., 308.}	68.0595	0.289942	newly 5 weeks cumul	982.	1285.22	{491., 2455.}	63.3147	0.220212	newly 49 weeks cumul	1514.	1730.93	{608., 3327.}	60.5636	0.239332
35	newly 3 weeks spec	115.	192.47	{83., 322.}	82.5692	0.214616	newly 3 weeks cumul	444.	763.348	{322., 1322.}	83.016	0.221584	newly 5 weeks spec	59.	108.636	{39., 204.}	100.411	0.234342	newly 5 weeks cumul	616.	1021.08	{417., 1764.}	79.9611	0.214112	newly 49 weeks cumul	837.	1329.97	{523., 2288.}	77.5135	0.209382
36	newly 3 weeks spec	124.	229.603	{90., 368.}	98.0159	0.239421	newly 3 weeks cumul	489.	951.825	{419., 1572.}	100.295	0.24505	newly 5 weeks spec	43.	123.365	{42., 187.}	190.07	0.349252	newly 5 weeks cumul	588.	1246.27	{525., 2013.}	116.443	0.267182	newly 51 weeks cumul	620.	1551.71	{626., 2465.}	151.173	0.314909
37	newly 3 weeks spec	61.	94.875	{19., 203.}	98.8934	0.303327	newly 3 weeks cumul	281.	475.875	{116., 1038.}	102.802	0.284205	newly 5 weeks spec	9.	33.15	{7., 72.}	279.444	0.472617	newly 5 weeks cumul	307.	568.875	{134., 1250.}	114.748	0.29154	newly 54 weeks cumul	480.	618.475	{146., 1361.}	82.2656	0.301
38	newly 3 weeks spec	169.	211.722	{77., 467.}	57.3197	0.210779	newly 3 weeks cumul	582.	866.944	{356., 1795.}	67.3444	0.210207	newly 5 weeks spec	127.	118.074	{34., 274.}	54.7098	0.270683	newly 5 weeks cumul	845.	1143.41	{458., 2438.}	59.6713	0.200221	newly 50 weeks cumul	1549.	1443.24	{556., 3134.}	48.5415	0.236489
39	newly 3 weeks spec	23.	166.591	{72., 307.}	624.308	0.779583	newly 3 weeks cumul	145.	686.477	{323., 1357.}	373.433	0.61902	newly 5 weeks spec	14.	93.	{33., 124.}	564.61	0.661112	newly 5 weeks cumul	176.	902.386	{411., 1702.}	412.72	0.639921	newly 51 weeks cumul	182.	1130.14	{475., 2047.}	520.954	0.698935
40	newly 3 weeks spec	69.	129.8	{61., 251.}	95.0725	0.253555	newly 3 weeks cumul	223.	423.467	{211., 708.}	93.8117	0.252462	newly 5 weeks spec	71.	106.867	{35., 233.}	79.3427	0.25174	newly 5 weeks cumul	369.	646.633	{300., 1220.}	85.664	0.240426	newly 24 weeks cumul	1107.	1464.03	{541., 2617.}	68.5125	0.23679
41	newly 3 weeks spec	7.	68.4474	{23., 111.}	877.82	0.894039	newly 3 weeks cumul	98.	388.474	{172., 591.}	296.402	0.525029	newly 5 weeks spec	0.1	22.0789	{6., 36.}	21978.9	2.22126	newly 5 weeks cumul	102.	449.421	{189., 696.}	340.609	0.569133	newly 55 weeks cumul	102.	479.237	{201., 719.}	369.84	0.595413
42	newly 3 weeks spec	113.	202.933	{80., 294.}	91.8977	0.247573	newly 3 weeks cumul	500.	776.2	{321., 1125.}	70.9111	0.217979	newly 5 weeks spec	90.	119.933	{40., 184.}	65.358	0.220107	newly 5 weeks cumul	679.	1050.6	{411., 1532.}	71.9817	0.219198	newly 47 weeks cumul	908.	1417.82	{564., 2075.}	74.4249	0.219667
43	newly 3 weeks spec	16.	170.674	{70., 288.}	966.712	0.930693	newly 3 weeks cumul	172.	766.217	{313., 1313.}	345.475	0.570846	newly 5 weeks spec	1.	74.4348	{27., 116.}	7343.48	1.75182	newly 5 weeks cumul	187.	954.13	{395., 1602.}	410.23	0.623488	newly 53 weeks cumul	187.	1091.89	{431., 1765.}	483.899	0.67542
44	newly 3 weeks spec	169.	314.545	{143., 589.}	94.4863	0.254106	newly 3 weeks cumul	606.	1140.45	{557., 1921.}	92.7843	0.254388	newly 5 weeks spec	127.	207.932	{77., 381.}	87.7774	0.24894	newly 5 weeks cumul	896.	1610.68	{747., 2791.}	88.1088	0.245767	newly 49 weeks cumul	1535.	2342.95	{937., 4095.}	73.8496	0.22207
45	newly 3 weeks spec	53.	193.813	{76., 378.}	266.097	0.451375	newly 3 weeks cumul	403.	957.672	{424., 1922.}	140.04	0.307514	newly 5 weeks spec	9.	72.2031	{24., 142.}	702.257	0.75774	newly 5 weeks cumul	435.	1149.11	{483., 2300.}	166.06	0.343709	newly 56 weeks cumul	452.	1253.72	{524., 2498.}	179.107	0.358938
46	newly 3 weeks spec	126.	235.545	{85., 403.}	96.7172	0.252799	newly 3 weeks cumul	521.	1015.73	{420., 1781.}	101.544	0.268078	newly 5 weeks spec	38.	111.75	{37., 195.}	198.744	0.396765	newly 5 weeks cumul	617.	1288.25	{522., 2245.}	114.885	0.289478	newly 53 weeks cumul	658.	1505.89	{617., 2626.}	134.018	0.317987
47	newly 3 weeks spec	195.	449.556	{223., 796.}	133.77	0.330058	newly 3 weeks cumul	736.	1728.43	{1003., 2827.}	136.561	0.344307	newly 5 weeks spec	94.	265.	{104., 507.}	185.54	0.388345	newly 5 weeks cumul	971.	2337.54	{1252., 3941.}	142.902	0.351553	newly 53 weeks cumul	1232.	3045.37	{1520., 5272.}	149.829	0.354686
48	newly 3 weeks spec	116.	273.227	{144., 490.}	139.224	0.329694	newly 3 weeks cumul	481.	1040.89	{622., 1747.}	120.473	0.31288	newly 5 weeks spec	50.	170.841	{67., 274.}	246.5	0.436456	newly 5 weeks cumul	630.	1429.39	{786., 2472.}	131.425	0.323378	newly 50 weeks cumul	758.	1913.25	{943., 3272.}	156.569	0.34587
49	newly 3 weeks spec	138.	166.243	{49., 359.}	65.5112	0.25545	newly 3 weeks cumul	440.	621.541	{209., 1311.}	71.3821	0.238896	newly 5 weeks spec	140.	105.351	{30., 245.}	65.1351	0.393055	newly 5 weeks cumul	714.	859.135	{292., 1875.}	63.54	0.247549	newly 46 weeks cumul	1855.	1187.54	{405., 2562.}	58.0054	0.383096
50	newly 3 weeks spec	69.	64.2632	{21., 117.}	47.7498	0.229577	newly 3 weeks cumul	243.	266.921	{102., 510.}	53.5954	0.22207	newly 5 weeks spec	43.	32.9211	{10., 66.}	57.4663	0.347274	newly 5 weeks cumul	357.	345.737	{121., 685.}	51.3047	0.239273	newly 46 weeks cumul	518.	438.263	{146., 906.}	52.6621	0.27594
